# Editorial: Training Intensity, Volume and Recovery Distribution Among Elite and Recreational Endurance Athletes

**DOI:** 10.3389/fphys.2019.00592

**Published:** 2019-05-21

**Authors:** Thomas L. Stöggl, Billy Sperlich

**Affiliations:** ^1^Department of Sport and Exercise Science, University of Salzburg, Salzburg, Austria; ^2^Integrative and Experimental Exercise Science and Training, University of Würzburg, Würzburg, Germany

**Keywords:** training intensity distribution, endurance diagnostics, training load and responses, high intensity training (HIT), polarized training, pyramidal training

Stimulated by the debate among endurance athletes and coaches of whether an optimal training intensity distribution (TID) exists, we recently reviewed the literature of studies dealing with TID in various sports (Stöggl and Sperlich, 2015; Stöggl, 2018). Our research identified numerous intensity zone models for quantifying TID. Among all TID models, a three zone-based TID seems widely applied among the literature. Within this model “Zone 1” represents low-intensity exercise below first lactate or ventilatory threshold. “Zone 2” exhibits accumulated levels of blood lactate between the first and second lactate or ventilatory threshold. “Zone 3” represents high-intensity exercise above the second lactate or ventilatory threshold.

Based on our (Stöggl and Sperlich, 2015) and other findings (e.g., Billat et al., 2001; Seiler and Kjerland, 2006; Sandbakk et al., 2011; Tonnessen et al., 2014) various TID exist including a so-called polarized (Zone1 > Zone3 > Zone 2) (Fiskerstrand and Seiler, 2004; Seiler and Kjerland, 2006) or pyramidal TID (Zone1 > Zone 2 > Zone 3) (Stöggl and Sperlich, 2015), depending on the discipline.

Since controlled and prospective training experiments are time consuming, complex, requiring both the coaches and athletes to adhere to scientific rules and methods we were motivated to stimulate this research topic to gain broader and deeper insights in TIDs during preparation, pre-competition, and competition phases in different endurance disciplines and performance levels. Ultimately, we wanted to identify TIDs demonstrating greater efficacy than others and highlight research gaps in an effort to direct future scientific investigations.

Based on the numerous contributions to this research topic we have learned the following:

## Method for Training Quantification

Based on Manunzio et al.'s analysis of 6-months preparation analysis of a 2nd place Race Across America finisher team (*n* = 4 athletes) the TID may vary depending on the method employed. Retrospective power data analysis based on the 3-zone model revealed a pyramidal TID (Zone 1: 63%; Zone: 28%; Zone 3: 9%) when including coasting phases (i.e., power output below 50% of power at maximum lactate steady state, MLSS). The same data set without coasting phases reflected a threshold TID with a greater Zone 2 emphasis (48/39/13%). The amount of training time < 50% of MLSS was shown to be remarkably high with 28% (104 h) of total training time.

## Power-Duration Relationship

In their concept (Hofmann and Tschakert) describe the relationship between power/velocity and the corresponding maximal duration/distance. Based on the power-duration relationship their concept may allow coaches or athletes to individually define the intensity and duration of an exercise session.

## Discipline Specific TID

In their attempt to answer the question of whether the training for marathon is harder than for an Ironman, Esteve-Lanao et al. employed the Objective Load Scale (ECOs) training load quantification method. For Ironman, the highest associations between performance and training were found with total training time and % of time in zone 1 (< Aerobic Threshold), while no association was found for total ECOs or amount of training in Zone 3 (>anaerobic threshold). Marathon performance was related to both total training time and total training load. Further, it was associated with total amount of Zone 1 plus the accumulation of any variable related to Zone 3 training. For both, Ironman and Marathon, the amount of Zone 2 training was associated with poorer competition performance. Ironman athletes trained more (more than twice total time, and >1/3 of training load) however less hard (e.g., training load per training hour) than marathon athletes. The differences in performance related to TID emphasizes the need for discipline specific TID analysis.

## Blocked TID

The study of McGawley et al. investigated the performance, stress, recovery and physiological effects to nine session of high intensity interval training (HIIT) evenly distributed over 3-weeks vs. nine HIIT sessions within 1 week with no further HIIT in the other 2 weeks. The authors showed that well-trained junior cross-country skiers are able to complete nine HIT sessions within 1 week without compromising total work done or experiencing greater stress or reduced recovery over a 3-weeks polarized microcycle. Although, both 3-weeks TIDs improved performance (i.e., 600-m roller skiing time trial) and various hormonal and muscle markers, the findings do not suggest that block-distributed HIIT is superior to evenly distributed HIIT within a 3-week period.

## Relation Between Training Load and Recovery-Stress State

Collette et al. aimed (i) to analyze the individual time-delayed linear effect relationship between training load and recovery-stress state with single-case time series methods and (ii) to monitor the acute recovery-stress state of high-performance swimmers over a 17-weeks macro cycle. The Acute Recovery and Stress Scale (ARSS), was shown to be a suitable tool for monitoring the acute recovery-stress state in swimming, especially with respect to the physical and overall scales, while the mental and emotional scales were not. Further, the authors recommend using the sRPE method, with respect to volume (km) rather than training time (h), to monitor the internal training load in swimmers.

## Gross Efficiency and TID

Skovereng et al. investigated the possible effects of initial performance, gross efficiency and VO_2peak_ on subsequent adaptations to a 12-weeks endurance training including HIIT (24 supervised HIIT sessions, 3/ and 1/weeks during recovery week in combination with *ad libitum* low intensity training) in competitive cyclists. In general, this training concept led to an increase in VO_2peak_, peak and mean power output during a 40 min time trial, while gross efficiency decreased. Initial performance demonstrated only small to moderate effects on training response.

## Sprint Interval Training (SIT) With Different Interval Recovery

In a 2-weeks experiment (6 sessions) by Olek et al. physically active males performed a series of 10-s sprints separated by either 1- or 4-min of recovery. The number of sprints progressed from four to six separated by 1–2 d rest. VO_2max_, citrate synthase activity and Wingate anaerobic test results improved similarly in both groups. Only end power output increased by 10.8% in the group with 1-min recovery. The two SIT protocols induced metabolic adaptations over a short period of time, and reduced recovery between SIT bouts may attenuate fatigue during maximal exercise.

## Seven-Day Running Block on Aortic Blood Pressure

Tomoto et al. investigated the impact of a condensed 7-day running camp on aortic blood pressure in two groups of collegiate endurance runners (i.e., one group accomplishing the weekly training target: 31 km/d vs. one group not accomplishing the weekly training target 13 km/d). Pulse wave analysis revealed elevated aortic blood pressure in the group with 31 km/d while this was not the case in the group with distinctly lower training volume and intensity.

## TID in Elite Female Cross-Country Skiers

The case study by Solli et al. presents the TID of the world's most successful female cross-country skier. Following a 12-years nonlinear increase in training load of approximately 80% (522–940 h/years) from the age of 20–35, the annual training volume during the five consecutive most successful years stabilized at 937 ± 25 h, distributed across 543 ± 9 sessions. She displayed a polarized TID, but to a lesser extent during the latter part of her career (i.e., 88/22/20% at the age of 20–27 years vs. 92/3/5% at the age of 28–35 years). While the total time in Zone-1 and 2 reduced from general preparation, to specific preparation and competition phase, Zone 3 increased from 4.1 to 9.2 sessions/month.

## Upper-Body Exercise

Børve et al. investigated the effects of replacing two HIT sessions with either combined upper-body muscular endurance training and running intervals (mixed endurance group) or only running intervals. Both concepts were performed as pyramidal TID. The 6-weeks mixed training approach increased both muscular endurance and maximal strength in a simulated double poling exercise and 1,000-m double poling performance following a 50-min submaximal trial with no changes in the endurance group. Specific upper-body muscular endurance training thus seems as a promising training model to optimize performance in well-trained cross-country skiers.

## Off-Training Analysis and TID

Not only the training stimulus itself but also other off-training stimuli may explain variation in adaptation among individuals (Sperlich and Holmberg), therefore it seems strange that the aspect of off-training behavior is mostly not considered within TID analysis. The study by Sperlich et al. demonstrated that national elite rowers demonstrate a substantial sedentary off-training behavior of more than 11.5 h/day. The question about the effects of off-training physical activity during recovery and the long-term performance development is open to future research.

## TID in Elite Rowers

Treff et al. analyzed different TID (polarized vs. pyramidal TID with similar amount of Zone 1 training) in national elite rowers. Based on their analysis both TID showed similar gains in performance. The polarized compared to the pyramidal TID seemed not to be superior, possibly due to a very similar percentage of Zone 1 training.

## TID and Acute Heart Rate Recovery and Anaerobic Power

Stöggl and Björklund explored whether four TID (9 weeks of HIIT vs. polarized TID vs. threshold vs. high volume low intensity) induced different responses on neuromuscular status, anaerobic capacity/power and acute heart rate recovery (HRR) in well-trained endurance athletes. They concluded that only a training regime that includes a significant amount of HIIT improves the neuromuscular status, anaerobic power and the acute HRR in well-trained endurance athletes.

## TID and Immune Function

Born et al. hypothesized that nine session of HIIT in 3 weeks would increase levels of salivary cortisol, reduce Immunoglobin-A secretion rate and impair mood thereby demonstrating marked psycho-immunological stress-response and compromised mucosal immune function compared to long slow distance (LSD) running. Based on their data the authors concluded that the increased Immunoglobin-A secretion rate with HIIT indicates no compromised mucosal immune function compared to LSD. Further, this shows the functional adaptation of the mucosal immune system in response to the increased stress and training load of nine sessions of HIIT.

Although the research topic broadened our understanding of various aspects of TID in elite and recreational sports, there are still various research question subject to future investigation, including the following (the authors are aware that this list is not exhaustive):

i) Currently most TIDs are defined as a certain percentage of training time or session within an intensity zone. However, this approach does not allow to judge the density of sessions nor the timing between sessions. Furthermore, depending on the sport strength training maybe included but differs substantially in intensity but no TID model, at least to our knowledge, has implemented this matter into a TID model.
ii) It remains unclear why (i) endurance athletes exercise a large proportion at low-intensity although endurance competitions are usually executed at higher intensity and (ii) why so many different TID may exist.
iii The long-term effects of TIDs (e.g., inverse polarized or HIIT) and potential shifting of TID within a season or between seasons are still not totally understood.
iv) We believe that the development of wearable technology should further ease and improve our understanding of different TID (e.g., the interaction of internal-external load, stress, fatigue and recovery process).

At this point we'd like to thank the authors for their contribution and we hope this research topic will not only provide new insights and viewpoints about the issues of TID in endurance training, but will also stimulate novel thoughts, experiments, and further advances in this field of research.

## Author Contributions

All authors listed have made a substantial, direct and intellectual contribution to the work, and approved it for publication.

### Conflict of Interest Statement

The authors declare that the research was conducted in the absence of any commercial or financial relationships that could be construed as a potential conflict of interest.
